# Perfusion Imaging and Inflammation Biomarkers Provide Complementary Information in Alzheimer’s Disease

**DOI:** 10.3233/JAD-230726

**Published:** 2023-11-21

**Authors:** Sofia Michopoulou, Angus Prosser, John Dickson, Matthew Guy, Jessica L. Teeling, Christopher Kipps

**Affiliations:** a University Hospital Southampton NHS Foundation Trust, Southampton, UK; b Clinical and Experimental Sciences, Faculty of Medicine, University of Southampton, Southampton, UK; c University College London Hospitals NHS Foundation Trust, London, UK; d Biological Sciences, University of Southampton, Southampton, UK

**Keywords:** Alzheimer’s disease, biomarkers, cerebrospinal fluid, inflammation, perfusion, SPECT

## Abstract

**Background:**

Single photon emission tomography (SPECT) can detect early changes in brain perfusion to support the diagnosis of dementia. Inflammation is a driver for dementia progression and measures of inflammation may further support dementia diagnosis.

**Objective:**

In this study, we assessed whether combining imaging with markers of inflammation improves prediction of the likelihood of Alzheimer’s disease (AD).

**Methods:**

We analyzed 91 participants datasets (Institutional Ethics Approval 20/NW/0222). AD biomarkers and markers of inflammation were measured in cerebrospinal fluid. Statistical parametric mapping was used to quantify brain perfusion differences in perfusion SPECT images. Logistic regression models were trained to evaluate the ability of imaging and inflammation markers, both individually and combined, to predict AD.

**Results:**

Regional perfusion reduction in the precuneus and medial temporal regions predicted Aβ_42_ status. Increase in inflammation markers predicted tau and neurodegeneration. Matrix metalloproteneinase-10, a marker of blood-brain barrier regulation, was associated with perfusion reduction in the right temporal lobe. Adenosine deaminase, an enzyme involved in sleep homeostasis and inflammation, was the strongest predictor of neurodegeneration with an odds ratio of 10.3. The area under the receiver operator characteristic curve for the logistic regression model was 0.76 for imaging and 0.76 for inflammation. Combining inflammation and imaging markers yielded an area under the curve of 0.85.

**Conclusions:**

Study results showed that markers of brain perfusion imaging and markers of inflammation provide complementary information in AD evaluation. Inflammation markers better predict tau status while perfusion imaging measures represent amyloid status. Combining imaging and inflammation improves AD prediction.

## INTRODUCTION

Diagnosis of dementia can be a long and iterative process which often requires multiple investigations including cognitive assessments, brain imaging, and cerebrospinal fluid (CSF) biomarker tests [1–3].

Early diagnosis in individuals with cognitive complaints facilitates early intervention and medical management which help optimize cognition, daily function, and quality of life for longer [4]. Early diagnosis reduces healthcare costs and residential support of patients living with dementia [5]. It also gives caregivers more time to adapt to their role, feel more competent to care for the patient, experience fewer psychological problems and delay institutionalization [4].

Neuroimaging modalities are already an integral part of the dementia diagnosis pathway [6] with functional neuroimaging techniques such as SPECT helping to identify subtle changes in brain perfusion to support diagnosis [7, 8]. Reduction of brain perfusion results in suboptimal delivery of energy, promoting neuronal disfunction [9], and perfusion SPECT is particularly valuable in the early stages of Alzheimer’s disease (AD) [10].

Beyond imaging, fluid biomarkers from blood and CSF have shown promise as new measures for AD diagnosis [11, 12]. One such group are measures of inflammation [13, 14]. As early as 2009, Holmes et al. showed that increased serum levels of proinflammatory cytokines were associated with faster cognitive decline [15]. Over the past two decades, an increasing body of evidence has emerged suggesting that neuroinflammation is the driving force behind neurodegeneration and dementia [16, 17]. Moreover, genome wide meta-analysis studies (GWAS) showed that genes associated with AD are linked to immune regulation [17–19]. These observations have led to the idea that neurodegeneration is driven by inflammatory mediators, which exacerbate the production of amyloid-β, the propagation of tau pathology and neuronal loss [16, 20, 21]. We recently confirmed that markers of inflammation in CSF correlate with tau CSF biomarkers and likelihood of dementia in a heterogenous clinical cohort [22]. Thus, markers of inflammation from blood and CSF may offer valuable diagnostic and prognostic information in AD diagnosis [23]. Beyond diagnosis, increasing our understanding of inflammation pathways could support identification of therapeutic agents with anti-inflammatory properties to inhibit tau aggregation and modify the course of AD [24].

In a recent commentary, Garibotto et al. suggested that molecular imaging and fluid biomarkers provide an opportunity for integrated diagnosis of AD. The combination of different sources of information could potentially provide additional diagnostic value by leveraging the strengths and weaknesses of both techniques [25].

In this study, we hypothesized that combining SPECT imaging with novel inflammation markers using regression models would improve diagnostic accuracy of AD. We aimed 1) to evaluate the association between inflammation markers and brain perfusion to define patterns of injury relating to neuroinflammation, 2) to evaluate the diagnostic accuracy resulting from the combination of imaging and inflammation biomarkers, and 3) to demonstrate this process in a real-world cohort.

We tested our hypothesis on a heterogenous cohort of patients referred to a memory clinic with cognitive complaints without exclusion criteria to provide a representative sample of a clinical population. To reduce subjectivity and potential influence of imaging and inflammation information on the final clinical diagnosis, in this study AD is defined biologically using the Paris-Lille-Montpellier (PLM) scale based on established thresholds for CSF measurements of Aβ_42_, Total Tau, and pTau [26–29].

## MATERIALS AND METHODS

### Patients and data

Data from participants with cognitive complaints suspected to be due to underlying neurodegenerative pathology were analyzed. Participants were referred to the Wessex Cognitive Disorders Clinic at University Hospital Southampton NHS Foundation Trust. Participants underwent brain perfusion SPECT scan and diagnostic lumbar puncture. Participants or their next of kin provided written informed consent and gave permission for storage of excess CSF. The data and samples were analyzed under the “Biomarker Research Assessing Inflammation in Neurodegeneration” (BRAIN) study with Institutional Research Ethics Committee approval number (NEU0383, REC 20/NW/0222). Inclusion criteria for the present study were 1) referral for cognitive complaints with query dementia, 2) availability of SPECT scan, 3) availability of CSF AD biomarker results, and 4) availability of excess CSF sample for inflammation analysis. No exclusion criteria applied, in order to evaluate the use of inflammation markers in a real-world, heterogenous cohort.

### Brain perfusion imaging

Participants were referred for brain perfusion scans as part of their clinical workup. Participants were administered 500MBq of Tc-99m HMPAO in a quiet room with low lighting. The SPECT scan started 15 min after the radiopharmaceutical injection.

SPECT imaging parameters were as following: low energy high resolution collimators, circular orbit with the radius minimized for each patient, 128 projections at 25 s/projection, energy window center 140 keV, window width 15%, matrix 128×128, zoom 1.45.

### Brain perfusion quantification

The SPECT scans were quantified using Statistical Parametric Mapping 12 (SPM12) [30, 31]. Each scan was registered to the MNI template and smoothed using a 16 mm Gaussian filter.

To enable group analysis of SPECT scans, the counts for each voxel of each individual participant’s scan were normalized using the cerebellum as the reference region. Additionally, age correction was applied based on a monoexponential fit of cerebellar counts derived from a database of healthy controls, as per standard clinical [33].

Participant specific t-score maps were created by comparing each individual scan to 31 healthy controls using SPM. Finally, the Marseille Region of Interest Toolbox [32] was used for extracting t-scores from regions that are well established for AD detection in brain perfusion imaging studies [33–35]. Specifically, precuneus and medial temporal lobe (MTL) regions, which were shown to be significant for predicting amyloid and tau abnormalities, were extracted using the volumes defined in the Automated Anatomical Labelling Atlas 3 (AAL3) [36].

### Measurement of inflammatory markers

A number of previous studies have looked at inflammation biomarker discovery over a broad range of proteins. Here we focused on four markers of inflammation that were recently identified as potential predictors of neurodegeneration [13, 22]. Levels of inflammation markers in CSF were measured by targeted proteomics analysis using the OLINK multiplex proximity extension array as described previously [22, 37]. CSF inflammation markers values were expressed in Normalized Protein eXpression (NPX) units in a log2 scale produced through data normalization. All samples were analyzed in the same batch and normalized to the same reference to enable comparisons across the patient cohort [38]. In this study, we focused on hepatocyte growth factor (HGF), a cytokine regulating inflammation and autoimmunity, matrix metalloproteinase-10 (MMP-10), an enzyme involved in blood brain-barrier function, tumor necrosis factor superfamily member 12 (TWEAK), a pro-angiogenic and pro-inflammatory cytokine, and adenosine deaminase (ADA), an enzyme involved in sleep homeostasis and inflammation.

### CSF AD biomarker measurement

Cerebrospinal fluid Aβ_42_, total tau, and phosphorylated tau levels were measured using chemiluminescent enzyme immunoassay by the UKAS accredited Neuroimmunology and CSF laboratory at UCLH as part of the participants standard clinical evaluation. Measurements were expressed as absolute concentrations in pg/ml. Kevashan et al. from the UCLH laboratory have previously reported on that measurements are comparable for a range of different measuring platforms [39].

Participants were classified as positive for Amyloid (A+), Tau (T+), or Neurodegeneration (N+) following the NIA-AA framework using predefined thresholds of CSF AD biomarkers of Aβ_42_, pTau, and total Tau, respectively, as outlined in Table 1 [27–29].

**Table 1:** CSF concentration thresholds for ATN classification

ATN Class	CSF concentration
A+	Aβ_42_ <680 pg/ml
T+	pTau >56 pg/ml
N+	Total Tau >355 pg/ml

Additionally, a simplified Paris-Lille-Montpellier (PLM) scale was used to provide a classification of the likelihood of a patient having AD. Patients with ≤1 positive biomarkers were classified in the low likelihood category (<25%), and patients with ≥2 positive biomarkers were classified in the high likelihood category for AD (>75%) [26].

### 2.6 Brain perfusion patterns of AD and inflammation

To visualize changes in brain perfusion patterns due to AD and inflammation markers, whole brain analysis was performed in SPM with individual markers added as covariates to univariate linear regression models. The following inflammation markers values were added as covariates: HGF, MMP-10, TWEAK, and ADA. Clusters larger than 50 voxels were extracted with (i) a family wise error (FWE) correction of *p* < 0.05, and (ii) and uncorrected test at *p* < 0.001.

### Statistical analysis and diagnostic models

SPSS Statistics (IBM v. 27) was used for statistical analysis. Pearson’s correlation coefficients were used to evaluate the association between the imaging regional t-scores, inflammation markers, CSF AD biomarkers, and age, with significant values highlighted at the *p* < 0.05 and *p* < 0.01 thresholds. Independent samples *t*-test was used to evaluate the impact of sex on imaging and CSF biomarkers, with significant values at *p* < 0.05 and Bonferroni correction for multiple comparisons.

Logistic regression models were trained to evaluate the ability of individual imaging and inflammation markers to predict which participants are positive for amyloid, tau, or neurodegeneration and to calculate the odds ratio for each marker. Additionally, logistic regression models were used to predict the participants’ likelihood of AD. Finally, receiver operator characteristic (ROC) curves were plotted to visualize the performance of the different markers in differentiating between low and high likelihood of AD as defined by PLM. Furthermore, the ability of imaging and inflammation markers to identify A+ from A–individuals was further assessed through logistic regression models and ROC analysis.

DeLong’s method was used to statistically compare the area under the ROC curves [40].

## RESULTS

Data from 91 participants were analyzed. 7 participants did not have total Tau values measured. Table 2 outlines the participants demographics, their classification as positive or negative for amyloid (A+/–), tau (T+/–), and neurodegeneration (N+/–) and the corresponding mean CSF concentrations of the corresponding AD biomarkers for each group. PLM scores characterize individuals with low, versus high, likelihood of AD pathology.

**Table 2:** Participant demographics, clinical classification, and CSF AD biomarker mean concentrations in CSF

	A+	A–	T+	T–	N+	N–	PLM Low	PLM High
Number of Participants	52	39	40	51	40	44	49	42
Age mean (SD)	68 (9)	66 (11)	65 (8)	69 (11)	66 (11)	68 (10)	68 (11)	66 (9)
Sex (F/M)	21/32	18/20	18/22	20/31	21/19	15/29	20/29	18/24
Aβ_42_ (pg/ml)	500	1086	591	877	668	861	926	546
pTau (pg/ml)	80.2	41.9	98.8	36.3	85.4	39.8	37.0	95.0
Total Tau (pg/ml)	686	346	838	325	843	250	304	837

### Association of inflammation markers and regional brain perfusion abnormalities with AD biomarker profile

The presence of a biomarker profile indicative of AD was significantly associated with measures of brain perfusion. Specifically, Aβ_42_, pTau, and total Tau values are associated with a reduction in brain perfusion in the precuneus region (*p* < 0.05), while Aβ_42_ alone correlated with MTL perfusion (*p* < 0.01). Levels of inflammation markers (HGF, MMP-10, TWEAK, and ADA) were significantly correlated with total tau and phosphorylated tau levels (*p* < 0.01), but not with amyloid levels. Brain perfusion in MTL was significantly correlated with precuneus, while the four inflammation markers were significantly correlated with each other (*p* < 0.01). MMP-10 was associated with reduction in perfusion in MTL (*p* < 0.05), while no other inflammation marker was associated with brain perfusion. Age was weakly but significantly associated with Aβ_42_ (*p* = 0.048) while its associations with other markers were not statistically significant. The correlation analysis results are outlined as a heatmap in Table 3. Independent samples *t*-test showed that TWEAK was significantly higher in female than male participants (*p* = 0.03), while none of the other biomarkers showed significant differences with sex.

**Table 3:** Heatmap of Pearson’s correlations for Imaging, Inflammation, AD biomarkers, and age

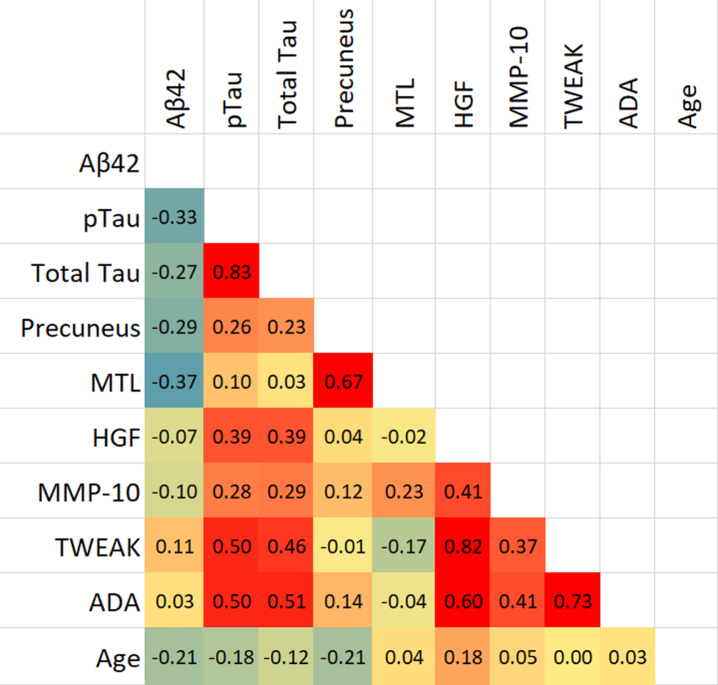

**Fig. 1:**
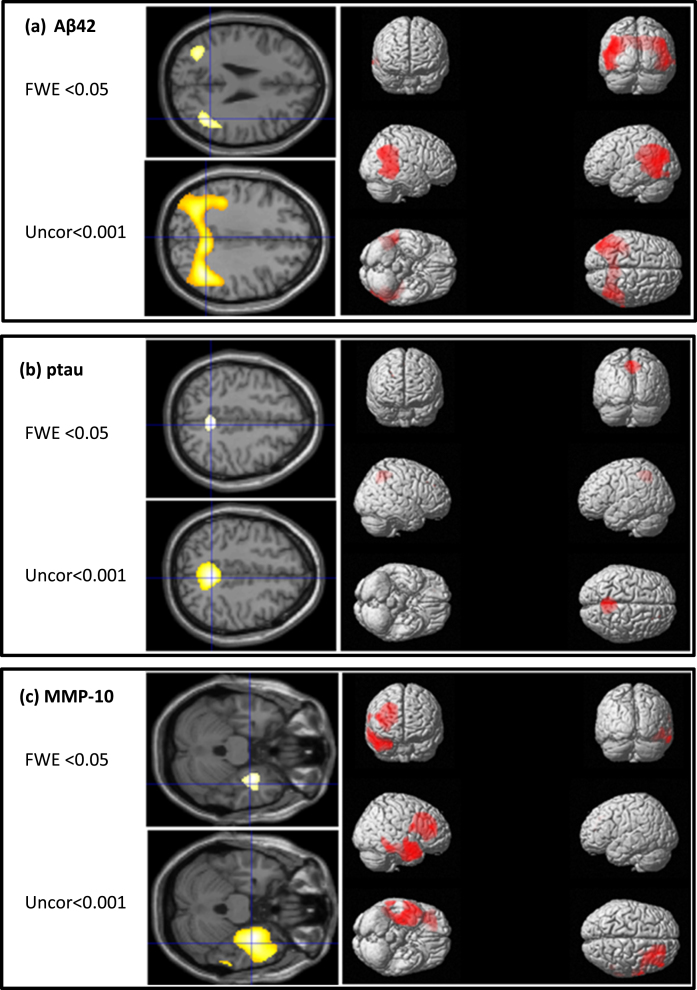
Overlays of clusters of hypoperfusion on MRI associated with changes in (a) Aβ_42_, (b) ptau, and (c) MMP-10 at FEW <0.05 and uncorrected <0.001 levels and surface rendering at the uncorrected <0.001 level.

CSF AD biomarkers and inflammation markers demonstrated distinct brain perfusion imaging patterns predictive of regional brain injury. Figure 1 illustrates changes in brain perfusion with Aβ_42_, pTau, and MMP-10 values. Decreased Aβ_42_ values (FWE, *p* < 0.05 and uncorrected *p* < 0.001) were linked to a significant reduction in perfusion in the parietal and temporal lobes bilaterally (cluster centers in angular gyrus and medial temporal lobes) based on whole brain analysis. Specifically, Fig. 1a shows significant reduction in perfusion in the parietal and temporal lobes, bilaterally (cluster centers in angular gyrus and medial temporal areas) with decreasing Aβ_42_ values.

Figure 1b reveals a significant reduction in medial parietal lobe perfusion (cluster center in precuneus) with increasing pTau values.

Finally, Fig. 1c, shows increasing MMP-10 values associated with significant reductions in perfusion in the right frontal and temporal lobes (cluster centers in medial frontal and parahippocampal areas). No significant perfusion clusters were identified for the other three inflammation markers.

### Inflammation markers are better predictors of tau and neurodegeneration status compared with regional perfusion abnormality

Both perfusion imaging abnormality and inflammation markers predicted individual CSF AD biomarker status. Table 4 presents the odds ratios for amyloid, tau and neurodegeneration based on univariate logistic regression models for individual imaging and inflammation markers. Perfusion reduction in the MTL increases the odds for amyloid positivity (OR 2.0). Increase in ADA results in higher odds ratio for neurodegeneration with a 10-fold likelihood increase (OR 10.3).

**Table 4:** Odds ratios (95% confidence intervals) for predictive Amyloid, Tau, and Neurodegeneration status based on univariate logistic regression models

	Amyloid	Tau	Neurodegeneration
Precuneus	1.7 (1.2 to 2.6)^*^	1.8 (1.3 to 2.8)^**^	1.7(1.3 to 3.1)^*^
MTL	2.0 (1.4 to 3.6)^**^	NS	NS
HGF	NS	5.9 (2.7 to 17.8)^**^	4.2 (1.8 to 12.1)^*^
MMP-10	NS	NS	NS
TWEAK	NS	6.0 (2.7 to 26.8)^**^	5.1 (2.2 to 18.1)^**^
ADA	NS	3.5 (1.7 to 13.6)^*^	10.3 (3.7 to 89.0)^**^

^*^Correlation is significant at the 0.005 level (2-tailed). ^**^Correlation is significant at the 0.001 level (2-tailed).

### Improved prediction of AD using combined imaging and inflammation variables in regression models

**Fig. 2:**
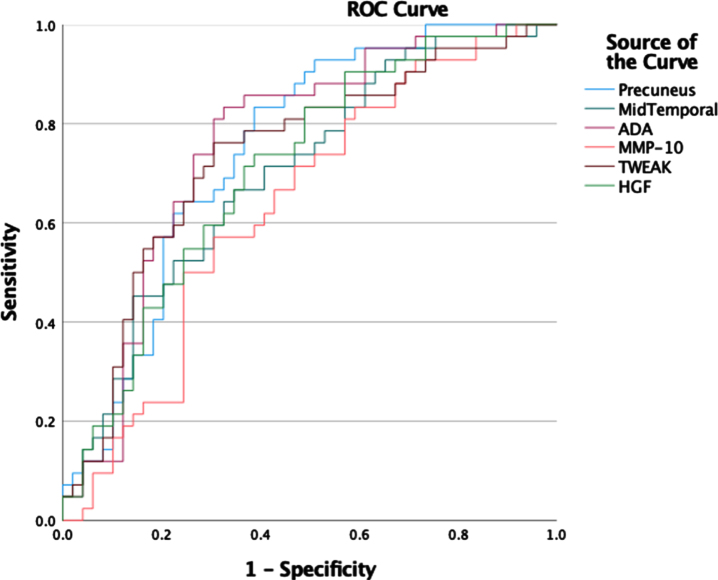
ROC curves for logistic regression classification of high versus low likelihood of AD using the individual imaging or inflammation markers.

**Fig. 3:**
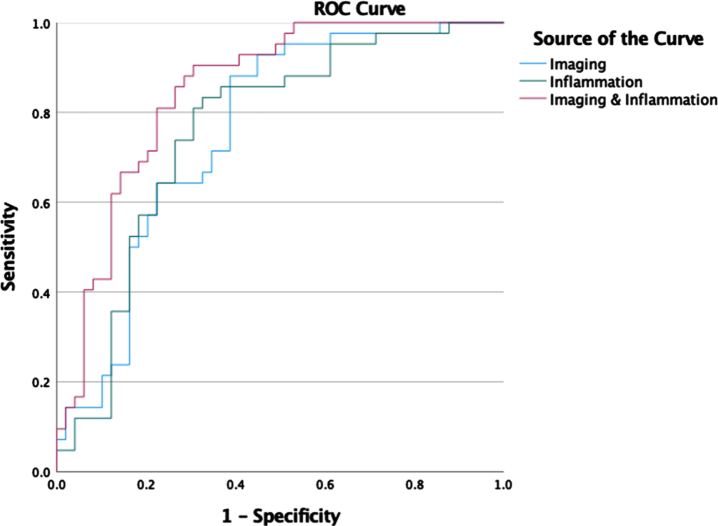
ROC Curves for logistic regression classification of high versus low likelihood of AD using imaging, inflammation, and combined markers (Precuneus, MTL, ADA).

Logistic regression models were trained to predict the likelihood of AD from imaging and inflammation markers. ROC curves for predicting high versus low likelihood of AD for each individual marker are outlined in Fig. 2. ROC for prediction of high versus low likelihood of AD for combined imaging and inflammation markers are shown in Fig. 3. To fulfil assumptions for logistic regression due to multicollinearity between markers and avoid calculating unstable regression coefficients, the combined model used only the Precuneus, MTL and ADA markers. The corresponding area under the curve values for the individual and combined markers are shown in Table 5. The statistical comparison of the ROCs showed that the combination of perfusion imaging and inflammation measures results in statistically significantly higher AUC than independently using inflammation (*p* = 0.035) or perfusion (*p* = 0.032).

**Table 5:** Logistic regression AUROC results for PLM derived likelihood of AD and identification of A+ status

Volume of Interest	AUROC PLM	AUROC A+
Precuneus	0.748	0.699
MTL	0.700	0.742
ADA	0.759	0.592
MMP10	0.635	0.643
TWEAK	0.734	0.545
HGF	0.708	0.627
Composite Imaging	0.758	0.747
Imaging and Inflammation	0.848	0.762

### 3.4 No change in prediction of amyloid positive status when combining imaging and inflammation variables in regression models

**Fig. 4:**
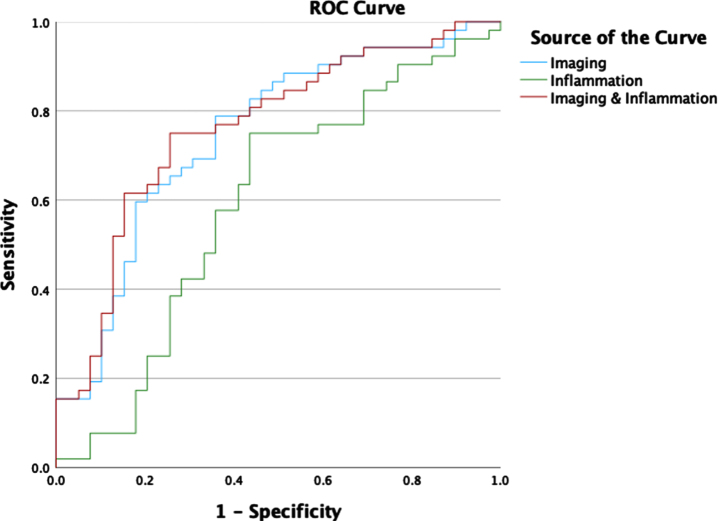
ROC curves for logistic regression classification of A+ versus A–participants using imaging, inflammation, and combined imaging and inflammation markers.

Logistic regression models were trained to predict the likelihood of A+ status from imaging and inflammation markers. ROC for prediction of A+ versus A–for combined imaging and inflammation markers are shown in Fig. 4. The corresponding area under the curve values for the individual and combined markers are shown in Table 5. Perfusion imaging is a better predictor of A+ status than CSF inflammation. The combination of inflammation and perfusion imaging markers does not provide a statistically significant improvement in the AUC compared to using imaging alone (*p* = 0.4).

## DISCUSSION

We show that CSF markers of inflammation and regional perfusion markers from brain imaging are both good predictors of AD. Analysis identified that temporo-parietal abnormality on imaging was predictive of amyloid status, whereas reduction in the precuneus, was predictive of tau status. The inflammation markers HGF, TWEAK, MMP10, and ADA, all showed significant associations with increasing levels of pTau and total Tau but not Aβ_42_ (Table 3). One of the markers of inflammation, MMP10, was associated with significant reductions in perfusion in the right frontal and temporal lobes. Combining regional perfusion abnormality and inflammation markers predicted AD biomarker abnormality (Aβ_42_, tau) more effectively than either alone.

Habert et al. have previously shown strong associations between pTau in CSF and precuneus perfusion reduction [41]. In contrast to our study, the study by Habert did not detect an association between Aβ_42_ and changes in perfusion. This may be due to the narrower range of CSF concentrations of Aβ_42_ in their study population, with mean Aβ_42_ 312 pg/ml for AD and 506 mg/ml aMCI, while our study cohort includes amyloid negative participants with mean Aβ_42_ 1086 pg/ml enabling evaluation of changes in perfusion along a broader spectrum of Aβ_42_ pathology.

Of the four markers of inflammation included in this study, an increase in ADA concentration in CSF was found to be the strongest overall predictor of neurodegeneration with an odds ratio of 10.3. ADA is an enzyme involved in the metabolism of adenosine, which impacts sleep homeostasis and increased levels have previously been detected in patients with chronic insomnia [42, 43]. ADA converts extracellular adenosine to inosine causing a shift of the immune balance towards a pro-inflammatory response [44].

Increase in MMP-10, a metalloproteinase involved in the breakdown of extracellular matrix, was associated with significant reduction in perfusion in the right temporal and frontal lobe (Fig. 1c). Matrix metalloproteinases are enzymes involved in blood-brain barrier function and increased levels have been associated with a more vulnerable blood-brain barrier in AD [45]. Blood-brain barrier breakdown results in a disruption of normal transport of nutrients and is implicated in both the onset and progression of AD [46].

A previous study by Bostrom et al. showed significantly increased levels of MMP-10 in patients with MCI progressing to AD compared to patients with stable MCI highlighting the potential of MMP-10 as a marker of progression [10]. Furthermore, Martino Adami et al. found faster cognitive decline in patients with increased MMP-10 levels. They demonstrated that inclusion of MMP-10 to the ATN scheme improves prognostic value [47]. The association with altered perfusion in the right medial temporal lobe identified in our study, further reinforces a role for MMP-10 as a marker of disease progression. The correlation with the right hemisphere may reflect differential impact of MMP-10 at different stages of disease [48].

A recent study by Liu et al. showed that reduction in brain perfusion is a sensitive marker for early amnestic MCI, which precedes volume loss [49]. As such, perfusion irregularities could be considered an upstream process in AD [9]. In our study reduction in brain perfusion measured by SPECT imaging was associated with increased levels of MMP-10 in the CSF. It is possible that perfusion deficits are a proxy for blood-brain barrier breakdown, which drives neuronal injury and failure of the neurovascular unit.

Despite the significant association of MMP-10 with brain perfusion SPECT imaging, the other three inflammation markers (ADA, TWEAK, and HGF) failed to show this relationship despite their strong associations with tau biomarker levels. This points to the possibility that these markers have differential roles with MMP-10 uniquely associated with structural and functional brain changes, a process that occurs relatively late in the pathophysiological process. Perhaps ADA, TWEAK, and HGF contribute directly to pathological changes that result in tau abnormality, while MMP-10 has a more significant role on the blood-brain barrier, altering perfusion in vulnerable brain regions detectable by perfusion SPECT imaging.

In our study we speculated that perfusion and inflammation are upstream of Aβ_42_ pathology in AD [9, 46]. While the results on perfusion are supportive of this speculation, our data does not support an upstream role for inflammation. In our study, as shown in Fig. 4, the inflammation markers do not contribute significantly to improving the prediction of A+ status provided by perfusion imaging. Instead, changes in inflammation are more likely downstream as they are associated with tau and neurodegeneration which reflect later changes in AD. Inflammation has been previously described as a mediator driving tau propagation and neurodegeneration, hence inflammation markers may be useful for evaluating the rate of progression [16, 20, 21, 23]. A specific example of this is increase in CSF levels of MMP-10 which was recently shown by Martino Adami et al. to be significantly associated with increased risk of progression to dementia [47]. Their study showed that this association occurs in the presence of abnormal CSF levels of Aβ_42_, further implying this marker of inflammation is downstream of amyloid. Finally, a recent Mendelian randomization study by Hansson er al. showed a causative link for CSF MMP-10 changes in preclinical AD.

Here, we hypothesized that combining imaging and inflammation markers would result in improved detection of AD. This conjecture is supported by the ROC analysis shown in Fig. 3 confirming an increase in classification performance suggesting each set of biomarkers contributes unique information to diagnostic classification.

Previous studies have highlighted the benefits of combining different types of biomarkers. Liu et al. reviewed diagnostic studies using multimodality imaging and non-imaging data from the Alzheimer’s Disease Neuroimaging Initiative studies and found that combining different measures yielded improved accuracy [50]. Habert et al. combined brain perfusion measures with Free and Cued Selective Reminding Test scores and found the combined measures performance exceeded that of imaging alone [51].

To our knowledge, this is the first study focusing on the combination of imaging perfusion with CSF markers of inflammation. Combining imaging and inflammation resulted in statistically significantly higher AUC than using imaging or inflammation alone. Our results suggest that integrated diagnostics [25] could add to predictive ability to detect changes leading to neuronal injury. Although this is unlikely to replace current evaluation with standard biomarkers (tau, Aβ_42_), the differential effects of these inflammation markers on AD pathology may help improve staging of disease, and in turn prediction of rate of progression and prognosis.

Our study has several limitations. The sample size is limited to 91 participants. No exclusion criteria were applied, and we cannot exclude the influence of mixed pathology, for example due to vascular pathology co-existing with AD. The clinical heterogeneity in this cohort serves as a real-world test for the imaging and markers of inflammation may provide beneficial measures when predicting disease. Despite the heterogeneity, both imaging and inflammation measures gave robust predictions of AD. Finally, this was a single-institution single-cohort study, and our findings warrant future validation with an independent cohort to evaluate generalizability beyond the Wessex Neurology Clinic data.

In conclusion, brain perfusion and CSF inflammation markers provide complementary information in AD evaluation and may support improved diagnosis and staging of disease. Future studies should include analysis of longitudinal cohorts to assess causal links between perfusion, inflammation, and AD. Furthermore, blood-based markers of inflammation should be considered as recent studies have shown reasonable correlation between CSF proteins and their plasma analogues, and blood-derived measures would enhance the diagnostic usability of inflammation markers in clinical practice [11]. Finally, imaging techniques that do not use ionizing radiation such as ASL MRI could be considered as alternatives to SPECT imaging going forward [52].

While ASL MRI is not currently included in clinical guidelines for AD and suffers from low signal to noise ratio and artifacts relating to hemodynamic factors [52–55], developments in MRI acquisition technology are helping to improve image quality and may support future use of ASL in AD evaluation [56, 57].

## Data Availability

For the purpose of open access, the author has applied a Creative Commons Attribution (CC BY) license to any Author Accepted Manuscript version arising. The samples and data were analyzed as part of the “Biomarker Research Assessing Inflammation in Neurodegeneration” (BRAIN) study with Institutional Research Ethics Committee approval number (NEU0383, REC 20/NW/0222). Due to research governance and ethical considerations, supporting data cannot be made openly available. Bona fide researchers may request supporting data by making a formal application to the corresponding author. The investigators will provide the data to any interested party subject to ethical approval.
